# Water hardness influenced variations in reproductive potential of two freshwater fish species; *Poecilia reticulata* and *Betta splendens*

**DOI:** 10.1186/s13104-020-05382-x

**Published:** 2020-11-19

**Authors:** Abarna Krishnakumar, E. S. Patrick Anton, Uthpala A. Jayawardena

**Affiliations:** 1 Department of Bio Science, Faculty of Applied Science, Vavuniya Campus, University of Jaffna, Vavuniya, Sri Lanka; 2 Department of Zoology, Faculty of Natural Sciences, The Open University of Sri Lanka, Colombo, Sri Lanka

**Keywords:** Water hardness, Freshwater fish, Fecundity, Gonadosomatic index, Growth performance

## Abstract

**Objective:**

Hardness of water in the form of CaCO_3_ affects reproductive potential in various fish species, differently. This study evaluates the effect of water hardness on growth and reproduction of two aquarium fishes, *Poecilia reticulata* (Ovo-viviparous sp.) and *Betta splendens* (Oviparous sp.) by growing them under 150 (control), 320, 540 and 900 ppm CaCO_3_ levels in semi natural aquaria.

**Results:**

Growth increased with increasing water hardness, reporting a significant progress of *P. reticulata* (p = 0.005) at 900 ppm. Similarly, the reproductive potential of *P. reticulata* was improved significantly, recording the highest fecundity (16.22 ± 3.90) and Gonadosomatic Index (GSI-2.48 ± 0.6) at 900 ppm. However, in *B. splendens* water hardness adversely affected the reproduction by resulting a significantly low hatchability and disturbed bubble nests at 900 ppm, compared to the largest bubble nest formed at the control condition (108.58 ± 16.19 cm^2^). Thus, the study revealed differential effects of water hardness on reproductive potential of the test species, by increasing the potential of *P. reticulata* while decreasing that of *B. splendens*. Though larval survival was affected in both species, larval growth was improved significantly in *P. reticulata* at 900 ppm level. Understanding reproductive potential of aquarium fishes in natural waters is crucial for their management purposes.

## Introduction

Water hardness i.e., a measure of calcium (Ca^2+^), magnesium (Mg^2+^) and/or iron (Fe^2+^) in water, is crucial for the growth, reproduction and embryo development of fish [1–4]. It affects oviparous and ovoviviparous fishes differently due to variations in their requirement of CaCO_3_ in the reproduction and growth [1, 5–8]. Considering the importance of hardness in the early life stage processes such as hatchability, larval growth and survival of eggs, it is recommended to maintain the water hardness above 20 ppm CaCO_3_ [9, 10].

Water hardness in Vavuniya District, of the Dry Zone of Sri Lanka reports remarkably high values, such as 900–1000 ppm [11]. In Vavuniya, ornamental fish trade is mainly dependent on ground water. Thus, evaluating the effect of higher hardness on fish health is of prime economic and ecological importance.

Freshwater species, *Poecillia reticulata* (Guppy) and *Betta splendens* (Siamese fighting fish) were popular verities in the aquarium fish trade. *P. reticulta* produces eggs that are hatched within the body (ovoviviparous, live bearers) and the hatchlings are born inside the female while *B. splendens* lay eggs in a foam nest (oviparous, egg layers) for external fertilization. Both species, can be easily reared and bred under laboratory conditions.

This study evaluates the effect of different water hardness on (i) the growth performance of adults and larvae, (ii) the reproductive potential and (iii) larval survival of two aquarium fish species, *P. reticulata* and *B. splendens* under semi-natural aquaria.

## Main text

### Methods

#### Preparation of aquaria

Glass aquaria of the size of 25 × 13 × 12 cm^3^ (24 tanks), simulating natural pond environment were used for the exposure. Being an aggressive fish, male *B.splendens* were kept individually in 20 separate cubic aquaria (12 × 12 × 12 cm^3^).

The experiment was composed of control (tap borne water 150 ppm CaCO_3_) and three treatment setups; 320, 540 and 900 ppm prepared by adding analytical grade CaCO_3_ to aged tap water. This series was selected to cover the hardness range, 100–1000 ppm in Vavuniya [8]. The hardness was determined by EDTA titration [12] and the treatment setups were screened weekly to maintain the relevant conditions. All the experiments were conducted as per the guidelines given by the research review panel of the Department of Bioscience, University of Jaffna.

#### Introduction of fishes to aquaria and maintenance

Healthy male and female fishes of *P. reticulata* and *B. splendens* were purchased from a nearby aquarium in Vavuniya and were transferred to the laboratory. Sexing was done by examining external morphology, where male fishes of both species possessed narrow and bright colored bodies and colorful caudal fins compared to round bellies and short caudal fins of the female fishes, displaying sexual dimorphism. Initial weight and standard length were measured, reporting 0.487 ± 0.008 g/2.98 ± 0.05 cm for *P. reticulata* and 1.266 ± 0.072 g/3.5 ± 0.01 cm for *B. splendens*. Then the fishes were acclimatized to aquarium condition (20 min. in each setup) and introduced to the experimental setups, 150 ppm (control), 320, 540 and 900 ppm CaCO_3_ and reared for 1½ month. In each experimental setup 15 females were introduced to 5 males of *B. splendens,* separately. For, *P. reticulata,* 25 females were introduced to 5 males. The exposure was conducted with three replicates.

Feeding (5% of the total body weight) was done twice a day at *ad libitum* with commercial fish pellets. The aquaria were maintained to keep the temperature 25–27 °C and pH 6.5–7.5 by replacing the media with newly prepared hard water on weekly basis. Debris was siphoned out. Mild aeration level was maintained as the fishes are air breathers.

#### Determination of growth performance

Weight and length of adult fish were measured at the sexual maturation, and the length weight relationship was analyzed, using W = a TLb: (Log W = log a + b log TL) [13] to obtain the linear regression.

#### Determination of reproductive potential

Female fishes showing gravid spots (Oocyte stage III–IV) (N = 6 per dose for each sp.) were randomly tested for fecundity (the number of ripening eggs found in the female just prior to spawning) and GSI (the ratio of fish gonad weight to body weight). Euthanizing was conducted with 0.02% MS222 solution.

#### Reproductive potential of *B. splendens*

To estimate the bubble nest size of *B. splendens*, three sets of clean breeding aquaria (size—60 × 30 × 30 cm^3^) were prepared without artificial bottom stones and aeration. A floating plant leaf was placed on the air water interface to facilitate the nest formation. After placing the male fish a clean glass cube containing a female fish was placed near the breeding aquarium to stimulate the nest building. Then the bubble nests built by male *B. splendens* (N = 5) were measured (diameter) by a ruler [14].

Hatchability of *B. splendens* was estimated after allowing a successful courtship with a gravid female. Without disturbing the bubble nest, the number of eggs released was counted. After a successful mating female was removed, and male was allowed for 24–48 h pre hatched parental care. Next, the hatched larvae were counted, and the hatchability was determined, as the number of larvae hatched over the total number eggs [15].

#### Reproductive potential of *P. reticulata*

Fertility of *P. reticulata* was determined by counting the intra-follicular embryos inside the female by sacrificing few fishes (N = 6) at the 21st day after mating [16]. The breeding tank for *P. reticulata* was formed with artificial aquarium stones and *Vallisneria* (a common water plant) to provide hiding place for the young ones. After the brooding parents were removed, and the hatchlings were counted.

#### Larval survival

Larval survival rate of both species (N = 20–30 per dose for each sp.) was determined after 1 week of exposure by counting the number of surviving one weeks-old larvae divided by the total number of hatched larvae/released young ones [15]. The larval growth performance (N = 12 per dose for each sp.) was estimated in every 10 days interval by measuring their length gain.

### Statistical analyses

Normality of the data set was tested before applying the statistics with SPSS 20.0 (IBM, USA).

Homogeneity and independence were tested for applying parametric analyses. One-way ANOVA and Tukey pairwise comparison were conducted to analyse the effects on weight, standard length, fecundity and Gonadosomatic Index, Laval growth (length) of the fishes. The linear regression analysis was used to find the length weight relationship (LWR). In LWR linear regression analysis, the slope of regression lines explicit the exponent coefficient value ‘b’. Variations in the estimates of ‘b’ for the fish species, examined from the expected value (ideal value ‘b’ = 3) were tested by t-test [17, 18]. Students t-test was applied to analyze the variation i.e. derived by dividing the difference between ‘b’ and ‘3’ by standard error of ‘b’ [19].

## Results

### Growth performance of exposed fishes

Growth improved with the increasing water hardness by showing significantly high weight values, particularly above 540 ppm treatments (p < 0.05), reaching 80% and 40% weight gains, respectively for *P. reticulata* (N = 30) and *B. splendens* (N = 20) in the highest hardness level (900 ppm). Similarly, length values of the both species were also increased with higher hardness levels, though only 14–17% elevations were recorded.

When the growth pattern was estimated *P. reticulata* showed isometric growth at 540 and 900 ppm levels while *B. splendens* showed isometric growth in all hardness levels. (Table 1).

**Table 1:** Growth performance and reproductive potential of *P. reticulata* and *B. splendens* under varying hard water treatment

	Growth performance			
	150 ppm (control)	320 ppm	540 ppm	900 ppm
Weight gain (g)				
* P. reticulata* (N = 30)	0.685^b^ ± 0.058	0.742^b^ ± 0.075	0.772^a,b^ ± 0.091	0.887^a^ ± 0.107
* B. splendens *(N = 20)	1.730^a^ ± 0.124	1.702^a^ ± 0.049	1.796^a^ ± 0.066	1.774^a^ ± 0.167
Length gain (cm)				
* P. reticulate *(N = 30)	3.25^b^ ± 0.03	3.31^b^ ± 0.04	3.31^b^ ± 0.05	3.49^a^ ± 0.02
* B. splendens *(N = 20)	3.77^a^ ± 0.20	3.93^a^ ± 0.31	3.92^a^ ± 0.24	4.01^a^ ± 0.31
Larval length gain (mm)				
* P. reticulata* (N = 12)	16.67^b^ ± 0.07	18.16^b^ ± 0.05	19.33^b^ ± 0.03	21.83^a^ ± 0.03
* B. splendens *(N = 12)	7.67^a^ ± 0.57	9.67^a^ ± 0.57	7.33^a^ ± 0.57	6.33^a^ ± 0.57

	Reproductive dynamics			
	150 ppm	320 ppm	540 ppm	900 ppm
GSI				
* P. reticulate *(N = 12)	1.35^b^ ± 0.39	1.38^b^ ± 0.54	1.50^b^ ± 0.66	2.48^a^ ± 0.60
* B. splendens *(N = 12)	17.01^a^ ± 1.15	15.03^a^ ± 2.61	17.25^a^ ± 4.41	15.83^a^ ± 1.33
Fecundity				
*P. reticulate *(N = 6)	8.80^b^ ± 2.59	9.00^b^ ± 3.54	9.80^a,b^ ± 4.32	16.20^a^ ± 3.90
*B. splendens *(N = 6)	743.3^a^ ± 83.3	605^a^ ± 179	824^a^ ± 175	694^a^ ± 272
Bubble nest (cm^2^)				
*B. splendens *(N = 5)	108.58^a^ ± 16.19	74.3^a^ ± 24.5	36.8^b^ ± 19	26.5^b^ ± 22.2
Hatchability				
*B. splendens *(N = 12)	78.1^a^ ± 17.6	85.72^a^ ± 3.52	29.02^b^ ± 10.32	24.89^b^ ± 3.42
Fertility				
*P. reticulate *(N = 12)	73.89^a^ ± 7.70	80.56^a^ ± 5.78	83.77^a^ ± 3.13	87.77^a^ ± 4.75

N values represent the number of fishes in each dose

^a,b^Value denoted by the same alphabetic superscript were not significantly differed from each other

### Reproductive potential of exposed fishes

Reproductive potential of *P. reticulata* showed significant variations in GSI, fecundity, bubble nest surface area, hatchability, fertility, under varying hardness levels. GSI of *P. reticulata* was increased with the hardness reporting a significantly higher value (Table 1, p = 0.016) at 900 ppm. However, GSI of *B. splendens* lowered though not significant with the treatment (Table 1, p = 0.731).

**Fig. 1:**
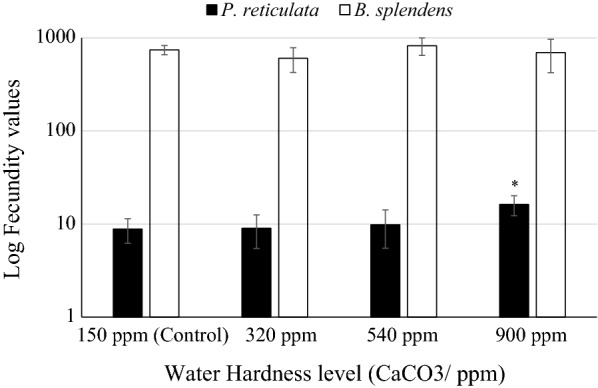
Logarithmic values of the fecundity of *P. reticulata* and *B. splendens* under varying water hardness treatments (150–900 ppm). (N = 6 per each dose for each species were used for the analysis). Error bars represents standard error of mean. Asterisk indicates the significant alteration from the control (p < 0.05, One way ANOVA, general linear model)

Fecundity of *P. reticulata* was increased with the water hardness, reporting a significantly higher value at 900 ppm. (16.20 ± 3.90, p = 0.016). Unlike the fecundity of *P. reticulata*, that of *B. splendens* was declined slightly by hardness treatment, showing only a slight elevation in 540 ppm (824 ± 175 eggs compared to 743 at the control, Fig. 1).

Bubble nest surface area of *B. splendens* showed significant decline along the hardness series, reporting 75% decline at 900 ppm (Table 1). In the hard water, 540 and 900 ppm, bubbles were blasted, and male fish was unable to rebuild the nest.

Fertility of *P. reticulata*, was increased along the hardness series, reporting 88% in 900 ppm compared to 74% at the control, though the increment was not significant (p > 0.05). Hatchability of *B. splendens* declined significantly in higher hardness levels above 540 ppm reporting only 25% success at 900 ppm compared to 78% in the control (p = 0.006). The percentage larval survival of both species declined gradually with the increasing hardness level, reporting significant declines above 320 ppm (p < 0.0009). However, the larval growth of *P. reticulata* was improved by higher hardness levels reaching a significant growth at 900 ppm (p = 0.006), though the effect on *B. splendens* larvae was insignificant.

## Discussion

*P. reticulata* and *B. splendens* displayed varying growth and reproductive potentials under different water hardness conditions. Growth (body weight and length) and the reproductive potential (gonadosomatic index, fecundity, bubble nest diameter, fertility) of *P. reticulata* were improved while most of those parameters were lowered in *B. splendens* with increasing hardness levels. These observations are compatible with previously reported studies for ovoviviparous and oviparous fishes, conducted elsewhere [20, 21].

According to Shim and Ho [20], dissolved Calcium is essential for growth of live bearer (ovoviviparous) fish especially *P. reticulata* as they are native to hard waters. They also found that rearing *P. reticulata* in extreme water hardness (2500 ppm) showed 10 times higher weight gain than in soft water (167 ppm). In the same way, James and Sampath [21] found that *Xiphophorus helleri* (live bearer) reared in 1018 ppm hardness level exhibited better growth performance compared to 76 ppm level. Water hardness influenced growth performances of *P. reticulata* were further reiterated by weight and length relationship, which revealed isometric growth occurred only above 540 ppm level. On the other hand, increasing water hardness showed no apparent effect on *B. splendens* resulting isometric growth in all hardness levels. Similarly, common snook *Centropomus undecimalis* and largemouth bass *Micropterus salmoides* exposed to higher hardness showed no considerable increase in growth parameters compared to their counter parts [3, 4].

Reproduction of *P. reticulata* was enhanced showing faster sexual maturation, in higher hardness conditions in compliance with James and Sampath [21] and Stratton [22], who reported higher and faster sexual maturation of *X. helleri*, in exceptionally high hardness medium. In line with this observation, Shim and Ho, [20] suggested that dissolved calcium is essential for sexual maturation of *P. reticulata*. As an oviparous fish, *B. splendens* showed slightly lowered reproductive potential under higher hardness levels. This is reiterated by previous work, Ratinam [8] who found suppressed gonadal development and maturity of *Pterophyllum scalare* and aborted maturation in *Barbus conchonius* and *Barbus letrazona* beyond 120 ppm hardness. Further, in compliance with previous studies, it was observed that high calcium in hard water deposit on the surface of the eggs of *B. splendens*, blocking the water absorbed into the perivitelline [7, 23] leading to dehydration and shrinking of the eggs [6]. Thus, being an oviparous fish *B. spledens* is suitable to the soft water than the hard water environment.

Increasing hardness caused high mortality in larvae of both species. Newborn are unable to tolerate the adverse environmental factors like extreme hard water [24] due to the stress condition in the physiology created by excess amount of calcium. Numerous studies carried on various fish species; *Clarias gariepinus*, Atlantic salmon (*Salmo salar*), rainbow trout (*Oncorhynchus mykiss*), and brown trout (*Salvelinus fontinails*), *Rutilus frisii kutum* (kutum), reiterated this finding [6, 7, 25, 26]. Thus, soft water is preferable for larval rearing for both *P. reticulata* and *B. splendens.*

Hence, it may conclude that *P. reticulata* requires more calcium for the growth and reproduction than *B. splendens* which grow and reproduce well in soft water environment. Besides their popularity in aquarium trade, *P. reticulata* and *B. splendens* are used as effective larvivorous species in the biological control of *Aedes aegypti* (dengue mosquito) in many parts of the world [27]. Thus, understanding their growth and reproductive potential in natural waters with varying hardness levels may provide valuable guidance to their integrated approaches.

## Limitations

This study was not intended to describe mechanism/s of action of CaCO_3_ in mediating growth and reproductive alteration of *P. reticulata* and *B. splendens*.

## Data Availability

The datasets used and/or analyzed during the current study are available from the corresponding author on reasonable request.

## Abbreviations

ANOVA: Analysis of variance
EDTA: Ethylenediaminetetraacetic acid
GSI: Gonadosomatic index
LWR: Length weight relationship
